# Shack-Hartmann Wavefront Sensing of Ultrashort Optical Vortices

**DOI:** 10.3390/s22010132

**Published:** 2021-12-25

**Authors:** Alok Kumar Pandey, Tanguy Larrieu, Guillaume Dovillaire, Sophie Kazamias, Olivier Guilbaud

**Affiliations:** 1 Laboratoire Irène Joliot-Curie, Université Paris-Saclay, UMR CNRS, Rue Ampère, Bâtiment 200, 91898 Orsay, France; tanguy.larrieu@ens-paris-saclay.fr (T.L.); sophie.kazamias@u-psud.fr (S.K.); olivier.guilbaud@u-psud.fr (O.G.); 2 Imagine Optic, 18, Rue Charles de Gaulle, 91400 Orsay, France; gdovillaire@imagine-optic.com

**Keywords:** shack-hartmann wavefront sensing, ultrashort optical vortices, orbital angular momentum, structured light

## Abstract

Light beams carrying Orbital Angular Momentum (OAM), also known as optical vortices (OV), have led to fascinating new developments in fields ranging from quantum communication to novel light–matter interaction aspects. Even though several techniques have emerged to synthesize these structured-beams, their detection, in particular, single-shot amplitude, wavefront, and modal content characterization, remains a challenging task. Here, we report the single-shot amplitude, wavefront, and modal content characterization of ultrashort OV using a Shack-Hartmann wavefront sensor. These vortex beams are obtained using spiral phase plates (SPPs) that are frequently used for high-intensity applications. The reconstructed wavefronts display a helical structure compatible with the topological charge induced by the SPPs. We affirm the accuracy of the optical field reconstruction by the wavefront sensor through an excellent agreement between the numerically backpropagated and experimentally obtained intensity distribution at the waist. Consequently, through Laguerre–Gauss (LG) decomposition of the reconstructed fields, we reveal the radial and azimuthal mode composition of vortex beams under different conditions. The potential of our method is further illustrated by characterizing asymmetric Gaussian vortices carrying fractional average OAM, and a realtime topological charge measurement at a 10Hz repetition rate. These results can promote Shack-Hartmann wavefront sensing as a single-shot OV characterization tool.

## 1. Introduction

Structured light beams have emerged as a versatile tool for a wide range of applications [1,2,3]. In particular, light beams manifesting Orbital Angular Momentum (OAM) [4] have propelled exciting new developments in the field of optical communication and quantum information [5,6,7,8], super-resolution microscopy [9,10], optical trapping and tweezing [11,12], material processing [13,14], astronomy [15,16], induction of topological current in semiconductors [17,18], and light–matter interaction [19,20,21,22]. These helically-phased beams, also known as optical vortices (OV), are characterized by their twisted wavefront resulting from an azimuthally varying phase given by exp(iℓϕ) around the beam propagation axis [4]. Such phase structure leads to an on-axis singular point with an undefined phase, followed by null on-axis amplitude, which ultimately yields a donut-like intensity distribution. The topological charge *ℓ* of the OV, also referred to as the phase winding number, designates the number of 2π shifts across the azimuthal coordinate ϕ in the transverse plane.

Over time, a wide range of techniques aimed at generating vortex beams have emerged [23,24]. However, the complete characterization of vortex beams still possesses numerous challenges. Common methods that are used to quantify the topological charge are based on interferometry with a reference beam [25,26,27], diffraction through apertures or gratings [28,29,30,31], employing cylindrical lenses [32], and exploiting dimensional properties of optical vortex beams [33,34]. Nonetheless, as these approaches are incapable of measuring both amplitude and phase, hence the complete field, they generally reveal only the average OAM value. Since the direct determination of the complex field is of paramount importance for all applications involving helical beams, these methods, therefore, are restricted in their utility. Most importantly, the techniques that are frequently used to impart a helical wavefront in the fundamental Gaussian beam are rather limited in their conversion efficiency, hence, often yielding a light field comprising superpositions of multiple helical modes [35,36,37,38,39,40]. Conclusively, full amplitude, phase, and modal content characterization of the OV become even more critical.

In the recent past, multi-shot iterative techniques have been used to reconstruct the amplitude and phase of the OV [41,42,43]. Nevertheless, these methods are prone to shot-to-shot beam fluctuations while being ambiguous to the absolute sign of the topological charge. In addition, multi-shot interferometric [44,45,46,47], and diffractive [48] methods have also been utilized for the amplitude, phase, and OAM spectrum characterization of vortex beams. Though these approaches enable high measurement precision and spatial resolution, they again suffer from stringent beam stability requirements. In [49], a single-shot interferometric alternative capable of retrieving the OAM spectra has been reported, which indeed minimizes the stability requirements. However, this method fails at simultaneous retrieval of OAM as well as radial mode content. Contrastingly, Shack-Hartmann and Hartmann wavefront sensing averts these issues [50,51,52,53], offering the possibility of single-shot simultaneous detection of amplitude and phase, even in the extreme-ultraviolet spectral range [52,53]. With regard to OV, the Shack-Hartmann wavefront sensor has previously been utilized to estimate the Poynting vector skews and topological charge from the measured slopes [54,55], ascertain the coherence characteristics of partially-coherent, and also few-cycle OV [56,57], and wavefront reconstruction of OV bearing a topological charge of ℓ=1 [58]. We also note that most of the recent works involving OV and Shack-Hartmann wavefront sensing have primarily focused on vortex position detection [59,60,61,62], and not the full amplitude and phase reconstruction. As the modal composition is embedded in the complex field of the light beams, a complete amplitude and phase characterization consequently provides much more information than just vortex position or Poynting vector skew angles. Conclusively, a convenient and reliable way to obtain the complete field of OV is highly desirable.

In this work, we demonstrate single-shot amplitude and phase characterization of femtosecond vortex pulses of topological charge up to ℓ=5 using a Shack-Hartmann wavefront sensor. These ultrashort OV are obtained using spiral phase plate (SPP). The reconstructed wavefront bears a helically twisting structure, which is an intrinsic characteristic of vortex beams. Furthermore, the overall twist of the reconstructed wavefront allows us to infer the topological charge of the generated beams. Subsequently, we establish the fidelity of our characterization approach through an excellent agreement between experimentally acquired and backpropagated intensity distribution at the waist utilizing the reconstructed field by the wavefront sensor. Through the spatial mode decomposition of the OV field into the orthonormal basis set of Laguerre–Gauss (LG) beams, we show that the beams generated by SPPs are in fact superposition of numerous azimuthal and radial modes. This allows us to assess the OAM purity, i.e., the energy contained in the desirable azimuthal mode for each topological charge. Following the above, we investigate the effect of radial clipping on the modal composition to demonstrate that radial aperturing primarily affects the radial mode distribution, while slightly influencing the overall OAM purity. For the ℓ=1 case, we extend our characterization to asymmetric Gaussian vortices that are obtained by axially displacing the SPP regarding the fundamental beam. The experimental results reveal that asymmetric Gaussian vortices manifest a fractional average OAM. Ultimately, we demonstrate realtime topological charge measurement at 10 Hz repetition rate over an extended period for OV of topological charge ℓ=1 to ℓ=4. These results demonstrate the potential of Shack-Hartmann wavefront sensors, which can promote their widespread usage for a complete characterization of OV.

## 2. Experimental Setup and Methods for Wavefront Characterization of Ultrashort Vortex Beams

**Figure 1:**
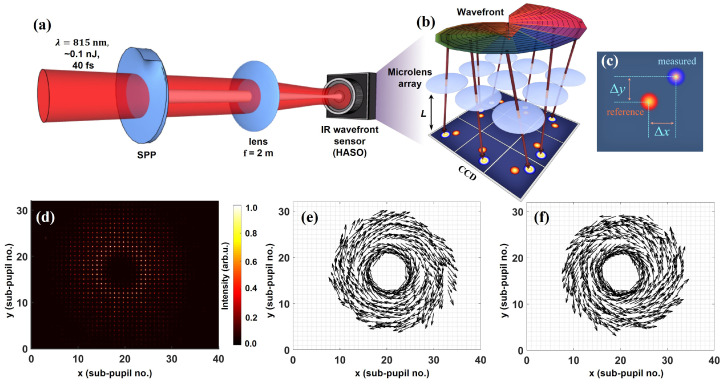
Vortex beam wavefront characterization setup. (**a**) Experimental setup for characterization of IR vortex beams. A low-energy ( ∼0.1 nJ) IR beam is passed through a spiral phase plate (SPP) to generate a vortex beam. The resulting beam is loosely focused by a 2 m focal length lens and directed towards a Shack-Hartmann wavefront sensor (HASO) located ∼300 mm after the focal plane. To generate a higher topological charge vortex, multiple SPPs of ℓ=1 are inserted in the beam. In the insets (**b**,**c**), we show the working principle of the Shack-Hartmann wavefront sensor. The incoming beam is sampled by the microlens array of the sensor. Depending on the local wavevector direction, the sampled beamlets are displaced concerning the reference spots of the calibrated wavefront sensor, as shown in (**c**). The sampled spot distribution is acquired on a CCD camera located at a given distance (L) from the microlens array. An example raw Hartmanngram is presented in (**d**) for a vortex beam of ℓ=4. The annular intensity profile of the vortex beam is evident from the central dark sub-pupils. From the raw Hartmanngram, the local slope, and ultimately the wavefront can be retrieved. In (**e**,**f**), the map of the measured slope for ℓ=+4 and ℓ=−4 are shown. The slopes maps show a spiraling behavior around the center, whose direction of rotation changes from clockwise (**e**) to counterclockwise (**f**) with a change in sign of *ℓ*.

The measurements were performed on a high-energy near-infrared femtosecond beamline that was primarily used to drive high-harmonic generation in rare gases under loose focusing geometry [63]. In Figure 1(a), we show the schematic of the experimental setup to generate and characterize the ultrashort optical vortices. A linearly polarized Gaussian beam of central wavelength 815 nm, pulse duration ∼40 fs, diameter ∼24 mm at $1/e^{2}$, root mean square (RMS) wavefront of ∼λ/32, ∼15 mJ maximum energy, and 10 Hz pulse repetition rate was passed through a spiral phase plate (SPP). The SPP modulated the phase of the incoming beam to impart helical wavefront. We used SPPs that were designed to match the central wavelength of the IR beam and offer transmission of ∼99.9% (HoloOr). For the OV of topological charge greater than 1, multiple SPPs were inserted in the incoming beam. Each phase plate was mounted on a 3-axis micropositioner stage, facilitating their precise alignment to the fundamental beam. For wavefront characterization, the energy of the incoming beam was scaled down to ∼0.1 nJ using a combination of half-waveplate and polarizer, and a series of beam samplers, each having a reflectivity of ∼10%. We remark that these reflective beam samplers allowed us to reduce the incoming vortex beam energy to the level supported by the wavefront sensor without using any transmissive elements, hence, with no or minimal effect on their wavefront. The resulting low-energy vortex beam was loosely focused by a 2 m focal length plano-convex lens and guided to a Shack-Hartmann wavefront sensor (HASO4 FIRST, Imagine Optic) placed ∼300 mm after the focal plane. The used sensor comprised a 32 × 40 microlens array with a spatial sampling of ∼110 μm, offering RMS tilt measurement sensitivity of ∼5 μrad and an absolute wavefront measurement accuracy of ∼λ/100 in the 750 nm to 850 nm wavelength range. We note that the main reason for using the focusing optics was to reduce the incoming beam diameter to match the pupil size of the sensor. For a beam of smaller diameter, usage of the focusing lens is not obligatory. Most importantly, the utilized sensor offered direct wavefront acquisition of converging and diverging F/5 beams with an accuracy of ∼λ/100 RMS, including astigmatism and high order aberrations.

### Optical Vortex Detection and Wavefront Reconstruction

The inset Figure 1b depicts the working principle of the Shack-Hartmann sensor for optical vortex wavefront characterization. The incoming vortex beam was divided into several beamlets by the microlens array, and their distribution was registered on a CCD camera located at a distance L from the sampling array. As the position of these beamlets on the CCD camera depends on the local wavevector direction, i.e., the derivative of the local wavefront, for each sub-pupil, the registered spots were dislocated by Δx and Δy with regard to the reference spots of the calibrated wavefront sensor. This is schematically represented in Figure 1c. In Figure 1d, we show a raw Hartmanngram for a vortex beam of ℓ=4. Remarkably, the central sub-pupils were unlit, indicating the presence of a singular structure that gave an annular profile typical to the vortex beams. Exploiting a centroid algorithm, the displacement of the sampled spots in the *x* and *y* direction ($\Delta x_{\left(i,j\right)}$ and $\Delta y_{\left(i,j\right)}$, respectively) for sub-pupil *i*, *j* was determined from the raw Hartmanngrams. From the displacements $\Delta x_{\left(i,j\right)}$, and $\Delta y_{\left(i,j\right)}$, the estimation of the local slope $S_{ij}^{x},\;S_{ij}^{y}$ along *x* and *y* direction is straightforward:

(1) $$S_{ij}^{x}=\tan\left(\frac{\Delta x_{\left(i,j\right)}}{L}\right)\approx \frac{\Delta x_{\left(i,j\right)}}{L}=\frac{\lambda }{2\pi }\frac{d\varphi (x_{\left(i,j\right)},\;y_{\left(i,j\right)})}{dx},$$

(2) $$S_{ij}^{y}=\tan\left(\frac{\Delta y_{\left(i,j\right)}}{L}\right)\approx \frac{\Delta y_{\left(i,j\right)}}{L}=\frac{\lambda }{2\pi }\frac{d\varphi (x_{\left(i,j\right)},\;y_{\left(i,j\right)})}{dy}.$$

In Figure 1e,f, we show the quiver plot of the slopes map of vortex beam corresponding to topological charge ℓ=+4 and ℓ=−4, where the positive (negative) sign indicates the clockwise (anticlockwise) rotation of the slopes, hence the wavefront. The length of the arrows is enlarged by a factor of 3 for better visualization, without affecting their orientation. Note that a global intensity threshold of 2% is applied to avoid the region where the signal level is too low to accurately determine the local slope. Noticeably, the measured slopes show a spiraling behavior around the center, indicating the presence of on-axis singularity. The measured slopes can either be used to extract the Poynting vector skew angle, as reported in [54], or to fully reconstruct the helical wavefront. Here, we restrict ourselves to the characterization of intensity and wavefront of these vortex beams. We note that for OV, the wavefront estimation from measured slopes is a nontrivial task, and different techniques have been proposed [58,64]. However, these approaches are computationally intensive and are inefficient for the reconstruction of dislocations of orders greater than four [58]. Therefore, instead of relying on these methods, we used a simplified approach that has successfully been used to reconstruct the wavefront of the vortex and vector-vortex beams exhibiting topological charge as high as 100 [65,66,67]. We purposefully introduced a discontinuity in the slopes map at an arbitrary position. The introduced discontinuity was chosen to be as small as possible, i.e., a discontinuity that requires ignoring the least number of sub-pupils. Additionally, the position of the discontinuity was optimized to yield a minimum peak-to-valley (PtV) wavefront value. In practice, the minimum PtV wavefront value was obtained when the introduced discontinuity was approximately perpendicular to the local slope. Following the above, the slopes were integrated using Southwell’s Successive over-relaxation (SOR) algorithm [68], implemented in proprietary software (WaveView, Imagine Optic), to reconstruct a 2-D phase map $\varphi (x_{\left(i,j\right)},\;y_{\left(i,j\right)})$. We emphasize that no presumptions were made on the structure of the wavefront during reconstruction. Integration of the slopes map with discontinuity directly resulted in all the wavefronts presented in this article. Even though this alternative does not reveal the absolute position of wavefront dislocation, as we show in Section 3.1, our method is indeed reliable. We also remark that a variation of less than 2% in the PtV wavefront value was observed for different positions of discontinuity in the slopes map, making our simple approach reliable for topological charge determination with a small error bar of 2% at most. Furthermore, owing to this simplified approach that is computationally less demanding, we demonstrate the realtime topological charge measurement at 10 Hz in Section 3.5.

## 3. Results

### 3.1. Intensity and Wavefront Characterization of Ultrashort Vortex Beams

**Figure 2:**
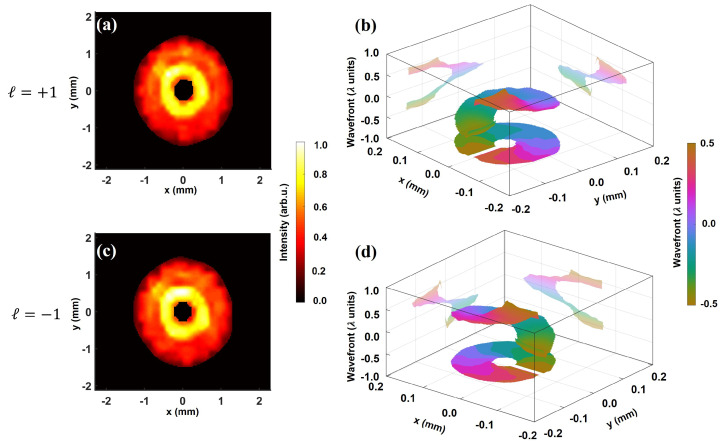
Characterization of low topological charge vortex beam. Reconstructed intensity (**left**) and wavefront (**right**) from single-shot raw Hartmanngram for beam of ℓ=+1 (**a**) and ℓ=−1 (**c**). The intensity distribution exhibits a dark central spot in both cases. The vertical axis in (**b**,**d**) represents the wavefront in the unit of wavelength λ. The wavefronts manifest a helical structure with an overall peak-to-valley value of ∼1λ, designating the unit topological charge. The handedness of wavefront rotation changes from clockwise (**b**) to counterclockwise (**d**) when the sign of the topological charge changes from positive to negative.

In Figure 2, we show the intensity and wavefront of the vortex beams of topological charge ℓ=+1 and −1. The wavefront is represented in the unit of wavelength. Note that the presented wavefronts are numerically corrected for tilt and quadratic curvature resulting from the focusing lens. Therefore, the displayed wavefronts account for all other residual aberrations present in the beam. Both for ℓ=+1 and −1, the intensity distribution exhibits an annular profile, typical for a vortex beam. Most strikingly, the reconstructed wavefronts show a helical structure with a smooth and continuous rotation by ∼1 wavelength, indicating a unit topological charge. In addition, the direction of wavefront twist that symbolizes the sign of the topological charge reverses from clockwise to anti-clockwise when comparing ℓ=+1 (Figure 2b) to ℓ=−1 (Figure 2d).

**Figure 3:**
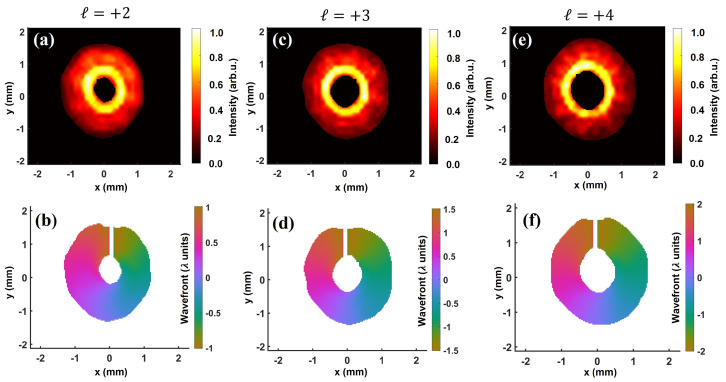
Characterization of vortex beams of higher topological charge. Intensity (**top**) and wavefront (**bottom**) for ℓ= 2 (**a**), 3 (**c**), and 4 (**e**). In all the cases, the intensity distribution presents an annular profile, whereas the wavefronts show a helical structure. The wavefront twists by ∼2.005λ, ∼3.003λ, and ∼4.0λ for ℓ= 2 (**b**), 3 (**d**), and 4 (**f**), respectively. The clockwise wavefront rotation indicates the positive sign of the topological charge.

Following the above, vortex beams with a higher topological charge are generated by inserting multiple SPPs in the Gaussian beam. The reconstructed intensity (top row) and wavefront (bottom row) for ℓ= 2, 3, and 4 are shown in Figure 3. In all the cases, the intensity distribution displays an annular profile. Additionally, the size of the annulus resulting from central phase singularity increases with the topological charge, as pointed out in [69]. Remarkably, for all the configurations, the reconstructed wavefront shows a smooth and continuous twist around the center. The total wavefront rotation, i.e., the PtV wavefront variation indicating the overall topological charge is within 1% of the theoretically expected value: ∼2.005λ, ∼3.003λ, and ∼4.0λ for ℓ= 2, 3, and 4, respectively. This demonstrates that the Shack-Hartmann wavefront sensing can be utilized to reliably reconstruct the helical wavefront of vortex beams of higher topological charge.

The complete characterization of the complex field of the vortex beams allows us to obtain the intensity and wavefront at the waist. In particular, the fidelity of wavefront reconstruction can be accessed through the relevance between the numerically backpropagated and experimentally obtained intensity distribution at the waist. We estimate the distance between the waist and the detection plane from the measured wavefront curvature radius. Thereafter, the reconstructed 2D complex field characterized by the wavefront sensor is numerically retropropagated to the waist using the Huygens-Fresnel integral.

**Figure 4:**
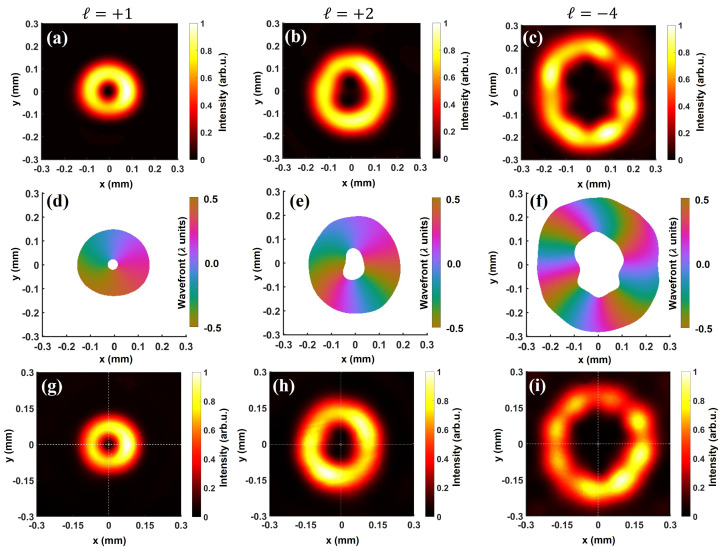
Fidelity of characterization: comparison between backpropagated and experimental intensity profile of vortex beams at the waist. We use the complex field reconstructed by the wavefront sensor to obtain intensity distribution (**top** row) and the wavefront (**middle** row) at the waist for ℓ=+1 (**a**,**d**,**g**), +2 (**b**,**e**,**h**), and −4 (**c**,**f**,**i**). The backpropagated wavefronts present a variation of 1, 2, and 4λ for |ℓ|=1, 2, and 4, respectively. For each case, the bottom row represents the experimentally obtained intensity profile at the waist. For all the configurations, the backpropagated and experimental profiles show an excellent agreement, indicating that the characterized amplitude and phase by the wavefront sensor indeed corresponds to the generated vortex beams.

For the selected cases of ℓ=+1, +2, and −4, we show the backpropagated intensity (top row), and wavefront (middle row) at the waist in Figure 4. For each condition, we also depict the experimentally acquired intensity distribution (bottom row) at the waist. The backpropagated wavefronts at the waist indeed exhibit a variation of 1λ, 2λ, and 4λ for |ℓ|=1, 2, and 4, respectively. Moreover, the direction of wavefront rotation also reverses with a change in the sign of topological charge. The intensity distribution at the waist shows an annular structure with a relatively larger central dark spot for a higher topological charge [69]. Most importantly, an excellent agreement between the backpropagated and experimental intensity profile for all the cases further demonstrates the merit of our characterization technique. The lack of ideal cylindrical symmetry and azimuthal modulation in the intensity distribution at the waist points towards the quasi-pure nature of the vortex beams generated using SPPs, which we will discuss in the following section. Furthermore, the presence of faint secondary intensity rings around the main ring indicates the radial mode character of the generated beams. In addition, these secondary rings are relatively more pronounced for the higher topological charges: comparing ℓ=+1,+2 (Figure 4g, h) and ℓ=−4 (Figure 4i). We further elaborate on this aspect in Section 3.2.

### 3.2. Modal Purity of the Vortex Beams

The characterized wavefronts reveal the topological charge as well as its sign. However, for applications such as quantum communication, information processing, and appropriate quantification of all light–matter interactions studies [19,20,21,22], including highly nonlinear frequency upconversion [70,71], it is imperative to deduce their full modal composition. Due to the Shack-Hartmann wavefront sensing, we can utilize the reconstructed complex field of the vortex beams to reveal their modal composition. The OAM spectra as a function of radial coordinate *r* can be obtained exploiting the Fourier-relationship between topological charge *ℓ* and azimuthal angle ϕ [72]. However, this approach does not reveal the radial mode content. On the contrary, decomposition of the complex field into the LG basis unveils both the azimuthal and radial mode content. In this section, we perform the modal analysis of the vortex beams by projecting the reconstructed field E(r,ϕ,z) by the wavefront sensor on a set of LG modes that form an orthonormal basis:

(3) $$E\left(r,\phi ,z\right)=LG_{\ell ,p}\left(r,\phi ,z\right)\;e^{ikz},$$

where

(4) $$\begin{array}{c} LG_{\ell ,p}\left(r,\phi ,z\right)=C_{\ell p}^{LG}\frac{w_{0}}{w(z)}{\left(\frac{r\sqrt{2}}{w(z)}\right)}^{\;|\ell |}\;\exp\left(-\frac{r^{2}}{w^{2}\left(z\right)}\right)L_{p}^{\left|\ell \right|}\left(\frac{2r^{2}}{w^{2}\left(z\right)}\right)e^{-i\ell \phi }e^{-ikr^{2}/2q\left(z\right)}\\ \times e^{i(\left(\ell +p\right)\;arctan\left(z/z_{R}\right))}. \end{array}$$

Here, *ℓ* and *p* are integers defining the azimuthal and radial mode indices, $C_{\ell p}^{LG}$ the normalization constant, $L_{p}^{\left|\ell \right|}$ is the generalized Laguerre polynomial, $z_{R}=\frac{\pi w_{0}^{2}}{\lambda }$ the Rayleigh length for beam waist $w_{0}$, $q\left(z\right)=z+iz_{R}$ the complex parameter of the modes, and $w\left(z\right)=w_{0}\;\sqrt{1+{\left(\frac{z}{z_{R}}\right)}^{2}}$. The normalization factor $C_{\ell p}^{LG}$ is chosen to satisfy the orthonormalization condition $\int_{0}^{\infty }\int_{0}^{2\pi }{LG}_{\ell ,\;p}\times {LG}_{\ell^{\prime },\;p^{\prime }}^{*}\;d\phi \;dr=\delta_{\ell \ell^{\prime }}\delta_{pp^{\prime }}$. The complex coefficient $c_{l,\;p}$ of the modal decomposition on the LG basis is then obtained by numerically calculating the following integral on the sensor sampling grid:

(5) $$c_{\ell ,p}=\int_{0}^{\infty }\int_{0}^{2\pi }E\left(r,\phi ,z\right)\times {LG}_{\ell ,p}^{*}\;d\phi \;dr$$

A set of orthogonal ${LG}_{\ell ,\;p}$ modes are fully defined by the choice of a common beam parameter *q*, i.e., by the distance z between the waist and the detector, and the waist size $w_{o}$ of the fundamental ${LG}_{0,\;0}$ mode [73]. Fortunately, both of these quantities can be retrieved from the Shack-Hartmann sensor data. On one hand, the distance between the beam waist and the detection plane is estimated from the wavefront curvature radius measured by the sensor. On the other hand, the waist size $w_{o}$ is deduced from the backpropagated intensity distribution at waist using [69]: $w_{o}=\sqrt{\frac{2}{\left|\ell \right|}}\;R_{max}$, where $R_{max}$ is the radius of the maximum intensity associated with the vortex beam of topological charge *ℓ*. Conclusively, wavefront sensing provides all the parameters needed for full modal analysis. In contrast to LG decomposition, the azimuthal mode analysis through Fourier transform of the measured complex field does not require the knowledge of beam waist size or position. In this case, the Fourier coefficient $c_{\ell }$ corresponding to topological charge *ℓ* at the detection plane can be calculated using:

(6) $$c_{\ell }={\left|\int_{0}^{+\infty }\;\int_{0}^{2\pi }E\left(r,\phi ,z\right)\cdot e^{-i\ell \phi }\;d\phi \;r\;dr\right|}^{2}.$$

**Figure 5:**
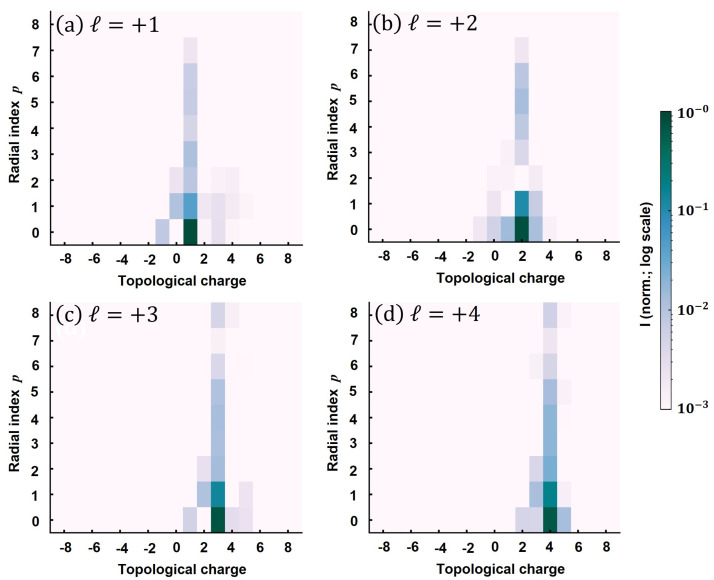
Modal analysis of the vortex beams. The ℓ,p modal content for (**a**–**d**). The global LG spectrum is normalized to unity. The contribution to the desired OAM mode is (**a**) ∼94%, (**b**,**c**) ∼93%, and (**d**) ∼92%, indicating high OAM purity in all the cases. The energy fraction contained in the dominant azimuthal and principal (p= 0) radial mode is: (**a**) ∼86%, (**b**) ∼79%, (**c**) ∼73%, and (**d**) ∼66%.

An important aspect of modal analysis is choosing a range of ℓ,p values that is sufficient to accommodate all the modes present in the beam. For the results presented herein, we consider the *ℓ* range in between −10 and +10, and the first 11 radial modes (p= 0 to 10), which account for more than 99.9% of the beam total energy. Subsequently, the complex weight corresponding to each mode is calculated. In Figure 5, we show the result of the LG modal analysis for the vortex beam of topological charge *ℓ* = 1 to 4. Note that the global LG spectrum is normalized to unity for easier comparison. Therefore, the presented spectra show constituent radial and azimuthal modes and their relative contribution to the total beam. Accordingly, the OAM purity, i.e., the contribution to desired azimuthal mode can be calculated by summing the energy fraction contained in the first 11 radial modes. On the other hand, the energy fraction in the first radial mode can be estimated by summing up the contributions corresponding to p= 0 mode in the stated *ℓ*-range.

In all the cases presented in Figure 5, the azimuthal mode purity exceeded 90%: ∼94% for ℓ= 1, ∼93% for ℓ= 2, 3, and ∼92% for ℓ= 4. The finite mode purity of the generated vortex beams arises from the fact that SPPs intrinsically are nonideal mode converters with limited conversion efficiency [36,37,38,39]. In particular, since SPPs are monochromatic by design, the topological charge dispersion naturally occurs for broadband (∼27 nm at FWHM, centered at λ= 815 nm) femtosecond pulses [38,39,44,74]. The modal purity is also reduced by the optical aberrations in the fundamental beam [75]. Even for an ideal monochromatic beam, the optical vortices generated by SPPs comprise a superposition of various LG modes [40]. Even so, the vortex beams show high OAM purity with only minor contributions to nonprincipal azimuthal modes. Meanwhile, all the beams possess a radial mode character, as evident from the contribution to higher *p*-orders. Though the OAM purity is just slightly affected by the increasing topological charge, the contribution to primary radial mode is drastically reduced for a higher *ℓ*: $LG_{\ell ,\;0}=$∼86% for ℓ= 1, ∼79% for ℓ= 2, ∼73% for ℓ= 3, and ∼66% for ℓ= 4. As SPPs are phase-only modulators, higher-order radial modes with the same OAM composition appear for a higher charge. This in turn diminishes the energy contained in the p= 0 mode, while only minutely affecting the overall OAM purity. Most importantly, the modal analyses of the experimental vortex beams reveal that the measured PtV wavefront indicating the overall topological charge does not necessarily imply 100% modal purity. Therefore, modal decomposition is mandatory to quantify the ℓ,p content of these beams.

**Figure 6:**
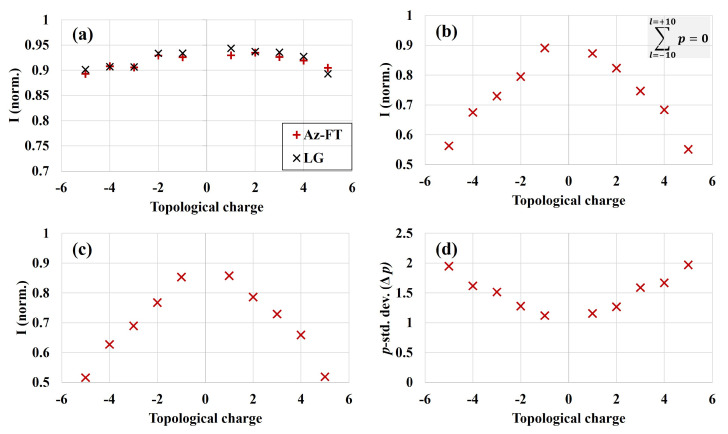
Modal analysis for the vortex beams of topological charge ℓ=−5 to +5. (**a**) Comparison of the OAM purity obtained via azimuthal Fourier transform and LG decomposition. (**b**) The energy contained in p= 0 radial mode summed over ℓ=−10 to ℓ=+10. (**c**) Contribution to $LG_{\ell =\ell ,\;p=0}$ mode. (**d**) The standard deviation of *p*-spectrum (Δp). The increasing trend of (Δp) with topological charge signifies a broader *p*-spectrum.

We note that the sign of the topological charge can simply be changed from positive to negative by flipping the side of the SPPs facing the incoming beam. In Figure 6, we show the result of modal analysis for vortex beams of topological charge ℓ=−5 to +5. A comparison between OAM purity obtained via azimuthal Fourier transform and full LG decomposition is shown in Figure 6a. Noticeably, both the approaches provided comparable results (<1% variation) with overall azimuthal mode purity remaining over ∼90% for all the topological charges. The OAM purity reduced from ∼94% for the lowest topological charge (ℓ=+1) to ∼90% for ℓ=+5. We point out that the given result can be also be interpreted as the OAM mode conversion efficiency of SPPs and their combinations, where a significant fraction of beam energy is contained in the desired OAM order. Since the higher azimuthal orders are obtained using multiple SPPs of unit topological charge, the small reduction in OAM conversion efficiency for higher *ℓ*-values might result from a slight misalignment of different phase plates with respect to the incoming beam, as pointed out in [76]. We further elaborate on this aspect in Section 3.4. Nonetheless, the generated beams still exhibited very high OAM purity. Figure 6b depicts the energy contained in the first radial mode (p=0) summed over a range of *ℓ*-values (ℓ=−10 to ℓ=+10), whereas the energy fraction in ${LG}_{\ell =\ell ,\;p=0}$ is presented in (c). Moreover, the standard deviation of radial mode distribution, which can be used to quantify the *p*-spectrum width (Δp), is shown in (d). In contrast to OAM purity that largely remained unaffected by the increase in topological charge, the contribution to the p= 0 radial mode was substantially reduced. For instance, in the case of ℓ=+1, ∼86% of total energy was contained in $LG_{\ell ,\;0}$ mode, whereas it dropped to ∼52% for ℓ=+5. As previously pointed out, because SPPs only modulate the phase of the fundamental beam, new higher-order radial modes with the same OAM are generated for a higher charge. In addition, the appearance of higher *p*-orders can also be inferred comparing the experimentally acquired intensity profiles depicted in Figure 4, where more pronounced secondary ring can be clearly seen for the higher topological charge beam. Additionally, a broader *p*-spectrum for the higher charge is evident from the increasing trend of Δp: almost two-fold increase when comparing ℓ=−1 to ℓ=−5 (Figure 6d).

In conclusion, the Shack-Hartmann amplitude and phase characterization, followed by modal decomposition, provides a deeper insight which is beyond simplistic techniques that rely on macroscopic diffractive, interferometric, or dimensional properties of OV. Commonly used methods that are unable to measure both amplitude and phase provide only the average OAM value and not their complete modal content. We also note that the Fourier transform method is relatively less computationally intensive than LG decomposition. Conclusively, owing to the excellent agreement between the two approaches (Figure 6a), Fourier transform decomposition can preferably be used for applications requiring characterization of only azimuthal mode content.

### 3.3. Effect of Radial Aperturing on Modal Content

**Figure 7:**
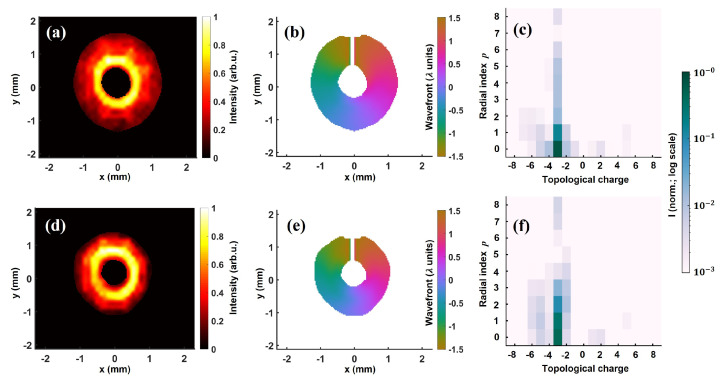
Effect of radial clipping on the modal content of ℓ=−3 vortex beam. (**a**) Intensity, (**b**) reconstructed wavefront in the unit of wavelength, and (**c**) modal content for unclipped beam. In (**d**–**f**), corresponding intensity, wavefront, and modal composition are shown for ∼16 mm iris diameter. The PtV wavefront signifying the topological charge is ∼3λ in both cases. The OAM purity, i.e., contribution to ℓ=−3 mode is ∼91%, ∼89% for fully open and 16 mm iris diameter, respectively. The contribution to $LG_{-3,\;0}$ mode plunges from ∼69% to ∼45% for the latter case.

Wavefront sensing also allows us to inspect the effects of different parameters on the modal composition of the vortex beams generated using SPPs. To analyze the consequence of radial clipping, an iris of variable diameter was placed in between the SPP and focusing lens (see Figure 1a). Thereafter, we performed intensity, wavefront, and modal content characterization for various iris diameters. In Figure 7, we show the intensity (left), reconstructed wavefront (center), and modal composition (right) obtained via LG decomposition for ℓ=−3 beam for fully open (top) and 16 mm (bottom) iris diameter. We remark that iris aperturing affects the size of the beam waist as well. Therefore, for each iris diameter, the beam waist $w_{0}$ has been accordingly redefined.

For both cases, an anticlockwise wavefront twist of ∼3λ indicated a −3 topological charge. The OAM purity was minutely affected by iris clipping: ∼91% for fully open and ∼89% for 16 mm iris diameter. On the other hand, the fraction of energy contained in $LG_{-3,\;0}$ mode sharply fell from ∼69% to ∼45% for the smaller iris diameter. This indicates that the radial clipping by the iris affects the modal distribution, in particular, the radial mode composition. Evidently, iris aperturing leads to redistribution of total energy to higher radial modes, which diminishes the contribution to the p= 0 mode. Note that unlike angular aperturing that broadens the OAM spectra due to uncertainty in the angular position and angular momentum [77], the radial clipping by the iris appears to affect primarily the radial mode distribution (comparing Figure 7c,f).

**Figure 8:**
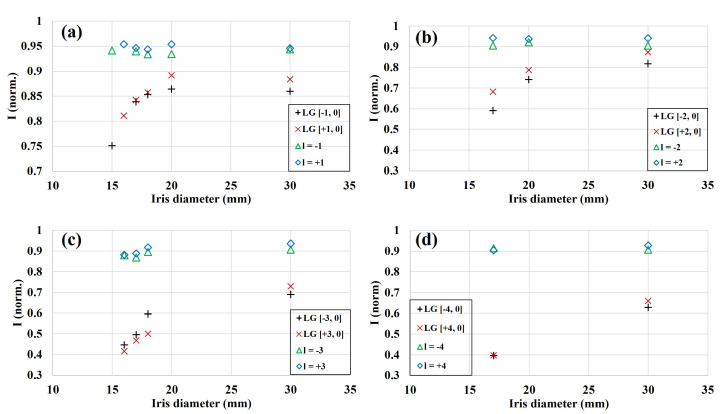
Effect of radial clipping on the modal content. The OAM purity and $LG_{\ell ,\;0}$ mode contribution for various iris diameters: (**a**) ℓ=±1, (**b**) ℓ=±2, (**c**) ℓ=±3, and (**d**) ℓ=±4. The 30 mm aperture diameter corresponds to a fully open iris. The contribution to desired OAM order only minutely varies with aperture size, whereas the energy contained in p= 0 radial mode shows a large reduction for the smaller diameter. This behavior is consistent for all the cases.

Though Figure 7 demonstrates this aspect for ℓ=−3 vortex beam, similar behavior was observed for other topological charges as well. Figure 8 represents the effect of aperturing on modal content for OV of ℓ=±1 to ±4. The general conclusion remains the same as for the ℓ=−3 beam (Figure 7). The OAM purity was slightly affected by aperturing. However, the contribution to the first radial mode reduced as the iris diameter became smaller. This trend was observed for all the configurations. Conclusively, Shack-Hartmann wavefront sensing indeed can be used as a reliable tool for intensity, wavefront, and modal composition characterization even for an apertured and quasi-pure vortex beam.

### 3.4. Effect of Spiral Phase Plate Displacement on the Modal Composition

The OAM of the optical vortices generated using SPP is sensitive to its alignment to the incoming Gaussian beam. The displacement of SPP generates an asymmetric Gaussian vortex [76], where the Gaussian and vortex beam centers are not superposed. Moreover, a shift of the Gaussian beam center from the center of the optical vortex can lead to the generation of asymmetric Gaussian vortex beams with fractional OAM [78]. Thankfully, wavefront sensing allows us to analyze the OAM composition of such beams. For the vortex beam of ℓ=+1, the SPP mounted on a 3-axis micropositioner stage was vertically displaced. Following the above, the beam was guided to the wavefront sensor for characterization.

**Figure 9:**
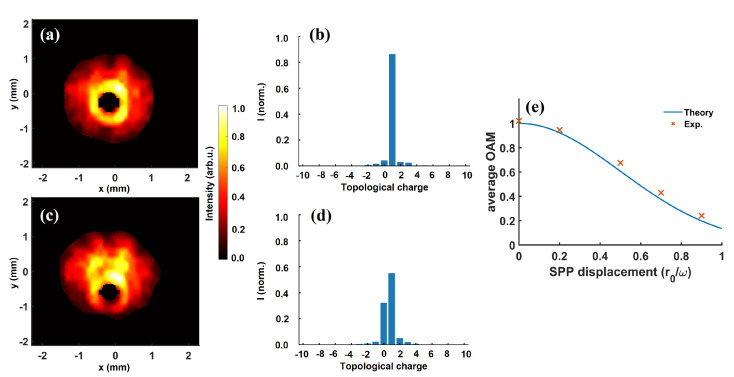
Effect of SPP displacement on the modal composition of ℓ=+1 vortex beam. Intensity (left) and OAM spectra (center) for SPP displacement $\alpha =r_{0}/\omega =0.2$ (**a**,**b**) and 0.5 (**c**,**d**). In (**e**), a comparison between theoretical (solid line) and experimental (cross symbol) average OAM for various SPP shifts is presented. The theoretical and experimental results are in very good agreement and indicate the fractional total OAM of asymmetric Gaussian vortices.

In the left column of Figure 9, we show the intensity profile for various SPP displacements, which is expressed as a ratio of the SPP shift $r_{0}$ and radius of the Gaussian beam ω. Figure 9a,c, displays the intensity profile for $\alpha =r_{0}/\omega =0.2$ and 0.5, respectively. In comparison to the centered SPP case (Figure 2a), we can see that the vortex center gradually drifted downwards from the center of the Gaussian beam. For α=0.5 (Figure 9c), the vortex center was already shifted in regard to the most intense part of the Gaussian beam. For further decentering, the vortex center continues to drift away from the center of the Gaussian beam. To observe the effect of SPP off-centering on modal composition, we utilized the reconstructed amplitude and phase by the wavefront sensor to retrieve the OAM spectra under these conditions. For the corresponding cases, the OAM spectra obtained via LG decomposition are presented in the second column of Figure 9. Remarkably, in comparison to centered SPP, the OAM purity already diminished from ∼94% (Figure 5a) to ∼86% for α= 0.2 (Figure 9b). For a further shift of α= 0.5, the contribution to ℓ=+ 1 mode sharply fell while the energy contained in ℓ= 0 order rapidly grows. For a greater value of α, this trend continues until most of the energy is contained in ℓ= 0 mode. As asymmetric Gaussian vortices are anticipated to carry a fractional average OAM, we show the experimental average OAM value (cross symbols) for various SPP shifts in Figure 9e. The OAM carried by the vortex beam exhibited a decreasing trend with SPP decentering. For α= 0.7, the average OAM was ∼0.45 i.e., a fraction of the average OAM in comparison to α= 0 case. To validate this trend, we compared the experimental result to the theoretical total OAM (solid line) for the asymmetric Gaussian vortex obtained using the analytical expression reported in [78]. The experimental and theoretical trends are in very good agreement, once again indicating the merit of experimental characterization. These results demonstrate the importance of proper alignment of SPP to the fundamental beam on one hand, and on the other hand, reveal the OAM composition of asymmetric Gaussian vortices. Conclusively, our wavefront sensing approach provides a direct insight into modal composition even in such an unconventional circumstance.

### 3.5. Topological Charge Measurement at 10 Hz

The maximum acquisition frequency of the used Shack-Hartmann wavefront sensor is 99 Hz. As the laser pulse-repetition rate was 10 Hz, the wavefront sensor provided an opportunity for realtime shot-to-shot amplitude and phase characterization of the vortex beams. For 2000 consecutive laser shots, we performed an extended measurement of the PtV wavefront value that signified the overall topological charge. In synchronization with the incoming beam, the wavefront sensor was externally triggered at 10 Hz.

**Figure 10:**
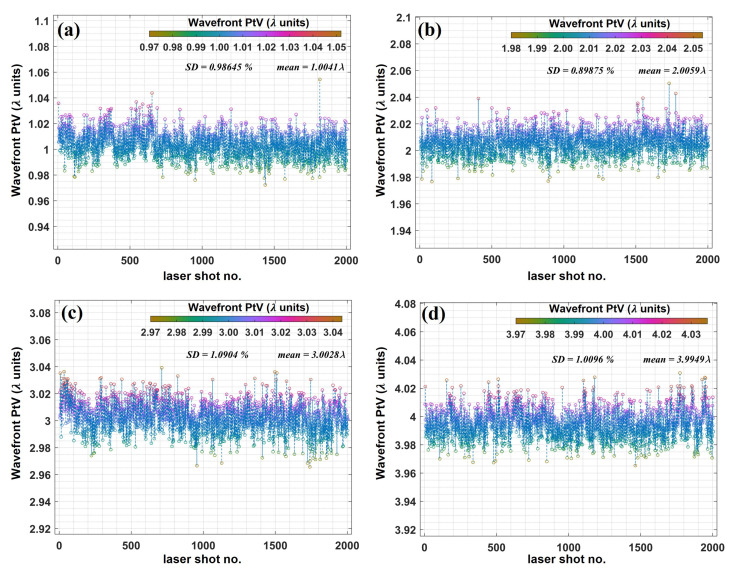
Wavefront twist measurement of femtosecond OV at 10 Hz repetition rate. Realtime PtV wavefront measurement over 2000 laser shots for (**a**) ℓ= 1, (**b**) ℓ= 2, (**c**) ℓ= 3, and (**d**) ℓ= 4. The mean PtV wavefront value and standard deviation (SD) of the measurement are included in the inset of each plot. In all the cases, the mean PtV wavefront indicating topological charge is within 0.5% of the theoretically expected value.

In Figure 10, we show the result of PtV wavefront measurement for vortex beams of ℓ= 1 to 4. The mean and standard deviation of the measurement is indicated in the inset of respective plots. Remarkably, the mean PtV wavefront values are within 0.5% of theoretical expectation: ∼1.004λ, ∼2.006λ, ∼3.003λ, and ∼3.995λ for ℓ= 1, 2, 3, and 4, respectively. Furthermore, the standard deviation of the measurement falls ∼1% or below for all the cases. Therefore, Shack-Hartmann wavefront sensing provides an opportunity to measure the topological charge, as well as its sign at a higher acquisition frequency. This, for instance, can be used for in situ amplitude and phase characterization of the low topological charge optical vortices that are often used to drive light–matter interaction and OAM nonlinear frequency upconversion processes. Incidentally, this also demonstrates the long-term stability of generated vortex beams.

## 4. Conclusions

We demonstrate the potential of a Shack-Hartmann wavefront sensor as a tool for single-shot characterization of femtosecond optical vortices. This diagnostic has been tested on an attenuated beam of a high-intensity laser facility, where the vortex structure is imprinted by a spiral phase plate. First and foremost, the slopes map, i.e., the measured wavefront tilts already show a spiraling behavior around the beam axis, which is the initial indication of the helical wavefront that is particular to optical vortices. As we later show, the reconstructed wavefront indeed presents a helical structure with an overall rotation falling within 1% of the theoretically expected value for all the cases investigated. The merit of our characterization is further reinforced by an excellent agreement between experimentally acquired and backpropagated intensity distribution at the waist exploiting the reconstructed field by the wavefront sensor. Proceeding with a Laguerre–Gauss mode decomposition of the field is therefore legitimate, and is a valuable tool to assess the azimuthal and radial mode purity. Even though the total wavefront twist confirms the topological charge, the modal decomposition reveals their quasi-pure nature. These minute modal imperfections arise from the fact that the SPPs are far from an ideal mode converter, consequently leading to a light field with superposition of multiple helical modes. We extend the modal characterization to radially apertured vortex beams. The experimental results reveal that radial clipping by a diaphragm mainly influences the radial mode distribution. The impact of radial aperturing on overall OAM purity is not as much as the energy contained in the first radial mode, which sharply falls for a smaller aperture. To further stretch the performance test of the wavefront sensor, for the beam of unit topological charge, we characterize the OAM content of asymmetric Gaussian vortex. The experimental results are in very good agreement with the theoretical predictions, confirming the fractional average OAM for such beams. Incidentally, this also shows the significance of proper axial alignment of SPP with respect to the Gaussian beam.

Ultimately, we demonstrate the characterization of the topological charge indicated by the peak-to-valley wavefront value at a 10 Hz repetition rate. As the wavefront peak-to-valley is a reliable indicator of the overall topological charge, this characterization method is ideal for realtime readout. Our work also shows that wavefront sensors can go beyond the rapid measurement of the vortex topological charge, giving access to their mode content in a single laser-shot. This aspect is particularly important for the appropriate quantification of all light-matter interactions involving optical vortices. Specifically in the context of high-intensity applications such as highly nonlinear frequency upconversion through high harmonic generation [70], highly nonlinear effects are expected to generate mixing between different helical modes present in the driver beam [71]. Conclusively, wavefront sensor-enabled single-shot amplitude, phase, and modal content characterization of ultrashort vortex beams driving these interactions can be a critical tool. Most importantly, the presented results demonstrate that a conventional Shack-Hartmann sensor, and a widely used zonal wavefront reconstruction method that is often used for Gaussian beams, can also be employed for optical vortices.

## Data Availability

All data needed to evaluate the conclusions are presented in the paper. Raw data can be obtained from the authors upon reasonable request.
